# Toxic Blood Hydrogen Cyanide Concentration as a Vital Sign of a Deceased Room Fire Victim—Case Report

**DOI:** 10.3390/toxics9020036

**Published:** 2021-02-16

**Authors:** Daniel Tabian, Gabi Drochioiu, Simona Irina Damian, Nona Girlescu, Oana Toma Gradinaru, Sebastian Ionut Toma, Diana Bulgaru Iliescu

**Affiliations:** 1 Faculty of Medicine, “Grigore T. Popa” University of Medicine and Pharmacy Iasi, 700115 Iasi, Romania; si_damian@yahoo.com (S.I.D.); nona.girlescu@yahoo.com (N.G.); bulgarudiana@yahoo.com (D.B.I.); 2 Faculty of Medicine, Transilvania University of Brasov, 500019 Brasov, Romania; 3 Brasov County Legal Medicine Service, 500073 Brasov, Romania; tomagradinaruoana@yahoo.com; 4 Faculty of Chemistry, “Alexandru Ioan Cuza” University of Iasi, 700605 Iasi, Romania; gabidr@uaic.ro

**Keywords:** hydrogen cyanide, carboxyhemoglobin, fire, smoke, postmortem, forensic toxicology

## Abstract

Carbon monoxide (CO) and hydrogen cyanide (HCN) are two common toxic products of combustion. HCN concentrations of fire victims are not routinely determined in most legal medicine services in Romania. We present the case of a room fire victim in which we evaluated the concentrations of HCN and carboxyhemoglobin (COHb), their contribution to the mechanism of death, and the possibility that HCN concentration can be interpreted as vital sign. COHb was determined by spectrophotometry. HCN was spectrophotometrically determined with ninhydrin in postmortem blood samples after its removal with 20% phosphoric acid and uptake into a solution of potassium carbonate. The presence of ethyl alcohol was determined by gas chromatography. The COHb concentration was 6.15%, while the blood HCN concentration was 1.043 µg × mL^−1^ and the total HCN was 1.904 µg × ml^−1^. A blood alcohol content of 4.36 g‰ and a urine alcohol content of 5.88 g‰ were also found. Although the fire produced a considerable amount of soot, and there were signs of inhalation of soot particles, the COHb level cannot be interpreted as a vital sign. Toxic concentrations of HCN and total HCN can be interpreted as a vital sign and indicates a contributive effect of HCN in the mechanism of death.

## 1. Introduction

The exposure to toxic smoke, alone or in combination with cutaneous burns, is responsible for up to 75–80% of deaths after confined space fires [1,2,3,4]. Carbon monoxide (CO) and hydrogen cyanide (HCN) are two common toxic products of combustion [1,4].

HCN is more likely to be encountered in fires in domestic structures with high temperatures and low oxygen content. This can be explained by the increased use of synthetic materials, containing higher levels of nitrogen, in interior finishing and furniture [1,2,3,4,5]. HCN liberation occurs when nitrogen-containing materials such as polyurethane, polyamide and polyacrylonitrile, wool, or silk are burned. These materials can be found in mattresses, pillows and upholstery, wooden furniture [6], victim’s clothes, plastic bottles, and other plastic household waste [5,7]. In addition, lethal concentration of HCN in air can be reached when 2 kg of polyacrylonitrile is combusted in a medium-sized living room [8].

The cyanide ion binds to the iron (III) of cytochrome a3 oxidase within mitochondria, inhibiting oxidative phosphorylation, which induces a shift to anaerobic metabolism, cellular ATP depletion, and lactic acidosis. Conversion to thiocyanate by the enzyme rhodanese and conversion into cyanocobalamin are physiologic detoxification mechanisms for low concentrations of cyanide. At toxic concentrations greater than 0.5 µg × mL^−1^, these mechanisms are overwhelmed, and signs and symptoms appear within seconds to minutes of exposure. A concentration greater than 3 µg × mL^−1^ is lethal. The effects of exposure to toxic or lethal concentrations of CO and HCN are synergistic. Whereas cyanide ions impair oxygen utilization at the cellular level, CO compromises arterial oxygen transport by combining with hemoglobin to form carboxyhemoglobin (COHb) and impairing tissue oxygen availability [2,5].

Toxic-to-lethal concentrations of HCN are often reported with high saturations of COHb. However, HCN’s role is still controversial, and HCN is difficult to detect both clinically and forensically because most hospitals do not have the necessary equipment to measure blood HCN concentrations [1,2,3,5]. The presence and diagnosis of CO poisoning in fire-related fatalities are well documented. Although acute HCN poisoning is now well recognized in some victims of domestic fire victims, to the best of the authors’ knowledge, it is currently not commonly determined in most legal medicine services from Romania and its exact incidence is unknown.

In the forensic assessment of a fire-related fatality, the question whether the victim was still alive when the fire started or the fire was set to remove traces of a prior murder is of crucial importance. In cases of bodies recovered from fire scenes, appropriate scene examination, appropriate autopsy, and toxicological investigation are essential to rule out competing causes of death. The most important signs of vitality come from soot deposits in the respiratory tract, the esophagus, and the stomach, suggesting smoke inhalation, as well as a COHb concentrations above 10% and the presence of hyperemic lines between the intact and burned skin [3,9,10].

Herein, we report a very unusual case of an indoor fire-related death. Although the fire produced a considerable amount of soot and there were signs of inhalation of soot particles, the COHb concentration raises a problem regarding the forensically relevant question whether the victim was still alive when the fire started. Moreover, our case indicates that testing for HCN is essential, and a toxic concentration of HCN in femoral blood is a sign that the victim must have been still alive when the fire began.

## 2. Materials and Methods

### 2.1. Background

The police investigation revealed a 51-year-old man, lying on the floor of the hallway of the house, coated with a greasy, brownish-black deposit of soot. The fire started in the night while the victim and his 77-year-old mother were inside the house. When the fire started, they evacuated the house, but then the victim entered the house again, trying to extinguish the fire with a wet coat. The body of the victim was discovered by the firefighters after the fire was extinguished. His mother said that the victim had consumed alcohol earlier during the day.

### 2.2. Autopsy

**Figure 1:**
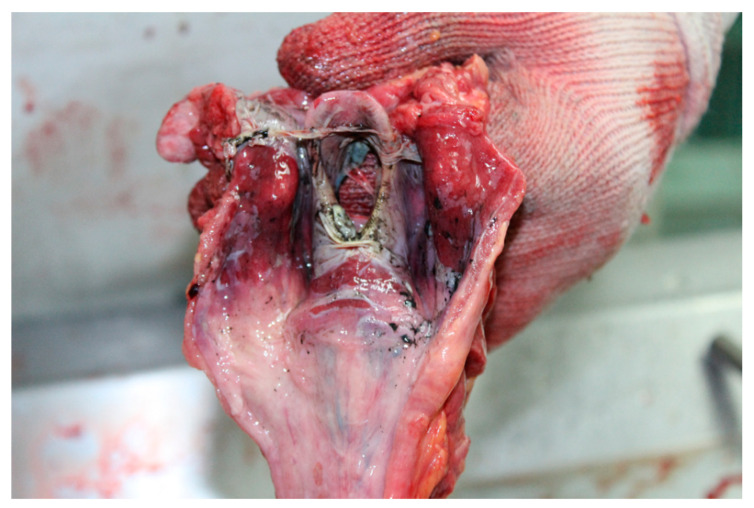
Laryngopharynx after dorsal dissection: The presence of deposited soot. Laryngeal edema and the effect of heat on tissues.

**Figure 2:**
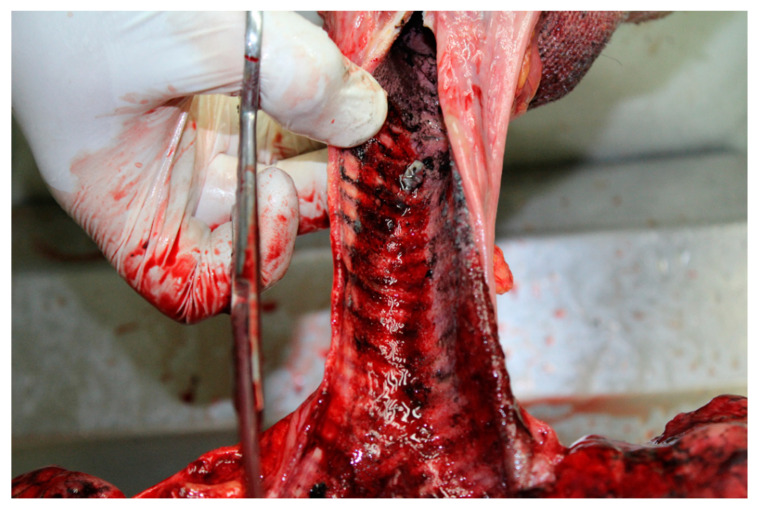
Trachea and the openings to the main bronchi after dorsal dissection: The presence of deposited soot.

The autopsy was performed 24 h after the death. External examination revealed dense, greasy, and brownish-black particles of soot on the body and bright red lividities. Second-degree burns on 50% of the body surface and hyperemic lines between the intact and burned skin were also observed. There were few scratches but no signs of external lethal violence. Internal examination revealed acute brain and pulmonary edema and acute glottic edema (Figure 1), with soot deposits on the upper and lower respiratory tract (Figure 2). Hypertrophied heart with a thickened myocardial wall and moderate coronary atherosclerosis were also noted.

### 2.3. Reagents and Procedure for the Determination of HCN and Thiocyanates

Analytical and statistical details of the method for determining HCN and thiocyanates (HSCN salts) in the blood were reported in a previous paper [7]. In brief, in order to determine the concentration of HCN and total cyanide compounds (total HCN), 5 mL of femoral blood was collected using a common syringe and placed in a test tube with sodium fluoride as an anticoagulant. The sample was stored at 4 °C, and the determination was made 48 h after the death. During inhalation of smoke containing HCN and CO, some of the HCN had been converted by rhodanese to HSCN salts [11]. Therefore, we also added 1 mL of 7.5 mg × mL^−1^ potassium permanganate (KMnO_4_) to each additional 1 mL blood sample before the treatment with 20% phosphoric acid (H_3_PO_4_). We adapted a microdiffusion method for HCN extraction using Conway cells and 20% H_3_PO_4_ solutions to remove HCN from the blood samples. In the inner chamber of a Conway cell, 2 mL of 2% potassium carbonate (K_2_CO_3_) (Merck, Darmstat, Germany) were pipetted to capture the gaseous HCN released from the outer chamber, where 1 mL of whole blood, 1 mL of ultrapure water, and 1 mL of 20% H_3_PO_4_ were added. The Conway cell was heated at 40 °C for 30 min on an electric heater with automatic shaker system (VMS-C10-2: VWR TM, Frankfurt, Germany). The temperature of 40 °C was chosen to avoid blood clotting. Then, the absorbent K_2_CO_3_ solution containing HCN captured and neutralized into potassium cyanide (KCN) was taken with an automatic pipette and introduced in a glass vial. The droplets found on the plastic lid of the Conway cell, which contained traces of HCN, were added to the absorbent solution and made up to 2 mL with distilled water.

After removing the 2 mL of cyanide-containing K_2_CO_3_, another 2 mL of 2% absorbent solution was added to the inner chamber of the Conway cell. Next, 1 mL of 7.5 mg × mL^−1^ KMnO_4_ solution was pipetted into the outer chamber to convert thiocyanates to HCN. Again, the Conway cell was heated at 40 °C for another 30 min, and the absorbent solution was adjusted to 2 mL.

**Figure 3:**
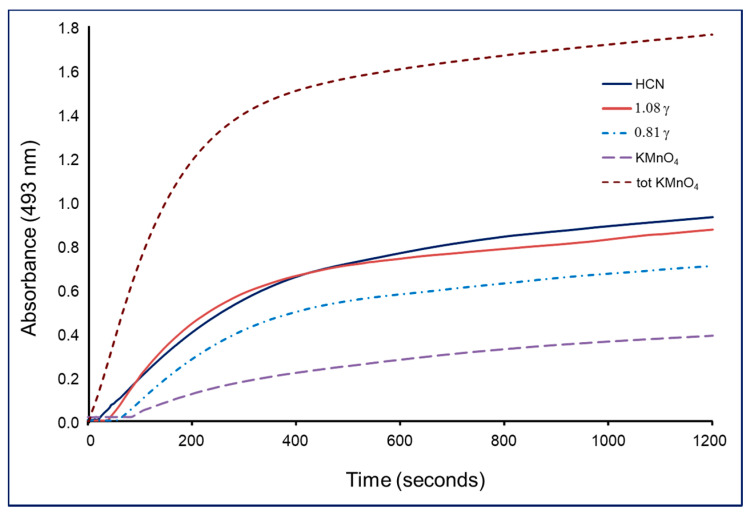
Kinetics of the reaction of cyanide with the ninhydrin reagent. Here, HCN—hydrogen cyanide removed first from blood with phosphoric acid; KMnO_4_—hydrogen cyanide resulting from the oxidation of thiocyanates in blood with potassium permanganate; tot KMnO_4_—total HCN resulting from one step treatment of 1 mL blood sample with potassium permanganate and phosphoric acid; 1.08 γ—1.08 µg × mL^−1^ KCN control solution; 0.81 γ—0.81 µg × mL^−1^ KCN control solution.

From each absorbent solution, aliquots of 100 µL were taken and treated with 1 mL of ninhydrin reagent (5 mg × mL^−1^ ninhydrin (2,2-dihydroxy-1,3-indandione from Serva, Heidelberg, Germany) in 2% K_2_CO_3_) in 1 cm-wide plastic cuvettes of a Libra S35 UV/Visible spectrophotometer. The kinetics of the color reaction between cyanide ions and ninhydrin reagent was monitored at 493 nm against a reference containing 100 µL of 2% K_2_CO_3_ solution treated with 1 mL of ninhydrin reagent (Figure 3). We compared the kinetic curves of both HCN removed from blood with H_3_PO_4_ and HCN from residual HSCN, which was liberated by the permanganate-phosphoric acid mixture, with those of two control KCN solution with known concentrations (0.81 µg × mL^−1^ and 1.08 µg × mL^−1^ respectively). In addition, we directly treated 1 mL of blood with the permanganate-phosphoric acid mixture and found higher absorbance values than the sum of those of free HCN and that from HSCN (Figure 3; tot KMnO_4_). We assumed that extracting HCN in two steps could cause some errors. Therefore, two Conway cells, one for free HCN and another one for total HCN, should be used to analyze the blood sample.

Moreover, HCN recovery from the blood samples varied from 75% to 85%, as determined with internal standards with known concentrations of KCN (0, 1.08, 1.625, 2.16 μg × mL^−1^ KCN). Therefore, when compared the absorbance values for HCN in blood samples with those from a calibration curve carried out with KCN solutions in the concentration range from 0 to 2.6 µg × mL^−1^, a blood HCN concentration of 0.75 µg × mL^−1^ and a total HCN one of 1.37 µg × mL^−1^ were calculated. However, we added 1 mL of 1.3 µg × mL^−1^ KCN to each blood sample as internal standard and thus determined the blood HCN more precisely. Finally, a blood HCN concentration of 1.043 µg × mL^−1^ and another of 1.904 µg × mL^−1^ for total hydrogen cyanide were determined.

### 2.4. Complementary Toxicological Tests

The COHb level was spectrophotometrically determined. In the reported case, the toxicological test carried out on a blood sample taken from the femoral vessels revealed the presence of COHb in a concentration of 6.15%.

The ethyl alcohol presence was determined by gas chromatography, the official method in Romania. The instrumentation used for analysis was an Agilent 6080 N (Germany) with an Agilent G 1888 headspace (Germany). The analytical columns used were a Restek Bac Plus 1 (USA) (30 m × 0.32 mm × 1.8 µm) and Restek Bac Plus 21 (USA) (30 m × 0.32 mm × 0.6µm). Helium was used as the carrier gas. All gases were ultrahigh purity. In brief, to determine the blood and urine alcohol content, 5 mL or femoral blood was collected using a common syringe and 5 mL of urine was collected by punction of urinary bladder. Then, 250 µL of sample was mixed with 1750 µL of internal standard (0.32‰ tert butanol) and placed in a 20 mL headspace vial. The vials were then crimp-sealed and placed on the instrument for analysis. All samples were analyzed on the instrumentation described above with a headspace oven temperature of 40 °C for 3 min and 30 °C/min to 100 °C for 1 min. The HS loop and transfer line temperatures were set at 90 °C and 110 °C, respectively. The GC cycle time was set at 13 min. Finally, toxicological examination on femoral blood and urine demonstrated a blood alcohol content of 4.36 g‰ and a urine alcohol content of 5.88 g‰.

### 2.5. Cause of Death

First, we considered that the cause of death was the severe second-degree burns on approximately 50% of the body surface, mixed with acute alcohol intoxication. However, relatively high levels of HCN in the blood and, in particular, the amount of HCN metabolized to HSCN, which provided evidence of a higher initial HCN concentration, suggest a possible cyanide poisoning as a contributive effect.

## 3. Discussion

The main results are summarized in Table 1. In the reported case of a fire fatality, the color of the lividity and the presence of soot particles on the body and within the respiratory tract (Figure 1 and Figure 2) suggest death due to smoke poisoning. Despite the fact that Popovic et al. (2009) reported a statistical significance between COHb saturation levels above 10% and the aspiration of soot, in our case, at 6.15%, the concentration of COHb was far below a level that would made CO poisoning plausible and cannot be interpreted as a vital sign of a smoker [10,12,13,14]. Büyük and Koçak (2009) conducted a retrospective study on 320 cases of fire burn-related fatalities. The authors reported that, in 12 of the accidental fire deaths, in spite of evidence that the deceased was alive when the fire started, there was no CO in the blood, which is in accordance to our data. The HCN level was not measured by Büyük and Koçak [10]. In point, in 2015, Ferrari and Giannuzzi reported the case of 33 inmates of a prison who died after their polyurethane mattresses were set on fire during an insurrection. For 9.3% of victims, the measured concentrations of COHb in blood samples were between 10.1–15.0%, while HCN concentrations were between 2.1–6.0 µg × mL^−1^ [15]. The unusual aspect of the case in study is the circumstance that although the fire produced considerable amount of soot, and there were signs of inhalation of soot particles, only a COHb concentration of 6.15% was determined.

From the cases reported in literature, it can be concluded that HCN is a potent and rapidly acting poison. HCN is likely to induce early incapacitation before the victim has time to inhale a significant CO dose. No relevant COHb concentrations were found in the corpses in these situations [2,3,9]. In a retrospective study with five fire victims with cardiac arrest, Kaita et al. (2018) reported that four (80%) victims had HCN concentrations above 0.5 µg × mL^−1^, which corresponds to significant exposure, and concentrations above 3 µg × mL^−1^ (suggesting a lethal dose) were found in three of the cases. Two of three patients who had a HCN concentration above the lethal level had COHb levels under 50%. The authors assumed that cardiac arrest might have been cyanide-induced [16]. In the cases reported by Stoll et al. (2016), concentrations of HCN between 0.5 µg × mL^−1^ and 2.0 µg × mL^−1^ and COHb levels between 15–40% were found in 12 of the reported cases [6]. The value under 10% which we obtained for the COHb concentration raises a problem regarding the forensically relevant question whether the victim was still alive when the fire began. Although the reported collection of findings is admittedly rare, our case illustrates that testing for cyanide is essential when HCN inhalation does occur [6]. In the presented case, the finding of a toxic concentration of cyanide in femoral blood was the sign that the victim must have been still alive when the fire began.

We can assume that HCN could have played a role in the mechanism of death in our case study. The incapacitating effect of exposure to HCN impairs the fire escape efficiency, such that the victim remains inside the room until heat exposure or toxicity results in injury or death. The asphyxiant effect, resulting in a shift from aerobic to anaerobic metabolism and lactic acidosis, determines neurological, respiratory, and cardiovascular depression. Confusion and loss of consciousness, bradycardia, hypotension, respiratory and cardiac arrest have also been described [2,3,16].

HCN is likely to be present in appreciable amounts in the blood of the victims of modern fires [17]. Our findings are consistent with those reviewed by Alarie [17], who examined the contribution of oxygen depletion and heat stress in fire deaths. He also considered the role of ethanol intoxication to be minor in such cases. The determination of blood alcohol is a routine analysis in all hospitals in Romania, and most of them use gas chromatography [18]. Although the high concentration of alcohol in the victim’s blood could not have been a cause of death, it could have prevented the victim from entering the burning house. Ethanol toxicity determines central nervous system inhibition and decreases excitation. Ethanol intoxication may determine impaired conscious level and disorientation, vomiting, tachydysrhythmias, and respiratory depression, followed by coma and even death. The appearance and severity of these symptoms depends on how quickly the alcohol is ingested and the peak of the blood alcohol concentration [4,14,19]. In the reported case, we can discuss alcohol tolerance due to the chronic use of alcohol, which would explain why, at the high levels of blood and urine alcohol concentrations, severe symptoms did not appear before the fire started. Furthermore, ethanol intoxication certainly contributed to the impaired ability of the victim to escape from smoke and fire, to the respiratory depression, and to death. In this sense, Büyük and Koçak reported in 2009 the presence of alcohol in the blood of the victim in 55 cases (17.2%) of 320 fire burn cases [10].

To determine the thiocyanates in blood, we combined a validated method based on the quantitative oxidation of thiocyanate with potassium permanganate at room temperature with the release of HCN, which reacts with picrate (developed by Haque and Bradbury in 1999), with another procedure based on the reaction of HCN with ninhydrin instead of picrat, advanced by Surleva and Drochioiu in 2013 [11,20]. Although Haque and Bradbury used 0.1–0.5 M (19.7–98.5 mg × mL^−1^ KMnO_4_) solutions of potassium permanganate and a reaction time of 3–16 h [11], we reduced the KMnO_4_ concentration to 7.5 mg ×mL^−1^ and the reaction time to 30 min at 40 °C without a significant decrease in the measured thiocyanate concentration. We also used internal standards with potassium thiocyanate and found a recovery rate of 75–85%.

In this paper, certain restrictions could have influenced the measurement of HCN. Baud et al. reported a decrease in the cyanide concentration after storage at 4 °C of 10.3 ± 2.2% after 1 day, and the decrease remained constant after 3 days (11.2 ± 2.1%) [8]. In our case, samples were collected 24 h after death and the determination was made 48 h after death. Taking these into account, one can presume that concentrations of HCN and total HCN were even higher. In their comprehensive study, Ferrari and Giannuzzi [15] asserted that blood samples kept at 3–4 °C remained fairly stable for weeks. The potential for cyanide to be produced postmortem is uncertain [8]. In another study, the authors affirmed that cyanide can be both produced and degraded in whole blood in vitro, but the phenomenon is not a constant. They found that cyanide disappearance from blood was maximal at room temperature and minimal at −20 °C, while cyanide production was maximal at −20 °C. They recommended that determinations be made immediately after the sampling [21].

Unfortunately, there have been some serious fires recently, and we have the opportunity to study some of them regarding the concentration of HCN in the blood of the victims (Piatra Neamt hospital and Matei Bals hospital in Bucharest). As for the Colectiv Nightclub fire (Bucharest, 30 October 2015), more than 64 people are believed to have died as a result of inhaling HCN from burning polyurethane foam, while more than 147 young people were injured [22,23]. We did not investigate this case, but our colleagues from the Polytechnic University of Bucharest made an evaluation of the composition of the air in the Collective Nightclub and found carbon monoxide and dioxide, nitrogen monoxide and dioxide, hydrocyanic acid, hydrochloric acid, toluene, and xylene. In addition, the manager of the Grigore Alexandrescu Hospital, Dan Enescu, declared that he did had not seen burns like the victims of the fire in Colectiv. Moreover, irritant components of fire effluents, having an instantaneous effect, can incapacitate fire victims, trapping them in a fire [22]. In addition, the longer term of the carcinogenic polycyclic aromatic hydrocarbons and microscopic particles are probably responsible for hundreds or thousands more deaths than the acute asphyxiants and irritants.

In some of the cases, the inhaled HCN appears to be the primary cause of death [2]. Moreover, a HCN concentration above 0.5 µg × mL^−1^ indicates significant exposure to smoke and can be considered as a vital sign [6,16,24]. Although HCN is increasingly recognized as an important asphyxiant, within the most legal medicine services in Romania, to the best of the authors’ knowledge, the concentration of HCN is not a routine test performed for fire-related fatalities and most of facilities do not have the possibility to perform the test. In the case in discussion, a toxic concentration of HCN and its metabolites was found, which can be interpreted as a vital sign and as a contributive effect in the mechanism of death. This gathering of findings, showing autopsy signs of CO poisoning, low concentration of COHb, and toxic levels of HCN and its metabolites, is considered extremely rare. This is a case report that presents a specific situation. It remains unclear whether our findings are generalizable to other cases of room fire victims. However, our discoveries in the presented case indicate the idea that the blood HCN level should be determined for victims exposed to smoke inhalation.

## Figures and Tables

**Table 1:** The main results of the presented case.

Parameters	Results
Age (years)	51
Sex	Male
Scene	Hallway of the house
Time to autopsy	24 h after death
Time to HCN determination	48 h after death
Autopsy findings	Bright red lividities, second-degree burns on 50% of the body surface, hyperemic lines, soot deposits on the respiratory tract
Blood level of COHb	6.15%
Blood level of HCN	1.043 µg × mL^−1^
Blood level of total HCN	1.904 µg × mL^−1^
Blood alcohol content	4.36 g‰
Urine alcohol content	5.88 g‰
The cause of death	The severe second degree burns on approximately 50% of the body surface, mixed with acute alcohol intoxication
The role of HCN	Possible contributive effect to death and vital sign
